# Quality check: ER-associated protein degradation and the control of grain size in rice

**DOI:** 10.1093/plcell/koad011

**Published:** 2023-01-18

**Authors:** Humberto Herrera-Ubaldo

**Affiliations:** Assistant Features Editor, The Plant Cell, American Society of Plant Biologists, USA; Department of Plant Sciences, University of Cambridge, Cambridge CB2 3EA, UK

Cells run continuous quality control checks on proteins. Defective, misfolded, or incompletely folded proteins are targeted for destruction by the Ubiquitin (Ub) proteasome pathway. The Ub-proteasome pathway participates in a wide range of physiological processes, including plant reproduction (reviewed in Linden and Callis, 2020). Thus, loss-of-function mutants of *GRAIN WIDTH AND WEIGHT 2* (*GW2*, encoding an E3 Ubiquitin ligase) exhibit abnormally wide grains (Song et al., 2007), while disruption of the gene encoding the deubiquitinating enzyme *UBIQUITIN SPECIFIC PROTEASE 15* (*OsUBP15*) promotes grain size and weight (Shi et al., 2019).

*In this issue of The Plant Cell*, the work by Jing Li, Baolan Zhang, and colleagues (Li et al., 2022) describes a role for the other ubiquitin proteasome system, the Endoplasmic reticulum-associated degradation (ERAD), in the regulation of grain size in rice.

A small grain mutant (*smg3-1*) was identified in an EMS-generated mutant population; *smg3-1* displayed shorter panicles and reduced grain number per panicle; the reduced grain size was found to be due to defects in cell expansion (see Figure). After the generation of an F2 population and sequencing, the authors identified a missense mutation in Os03g19500, a locus encoding a ubiquitin-conjugating enzyme. A wild-type genomic transgene of Os03g19500 rescued the defects in grain size of the *smg3-1* mutant. Two CRISPR alleles of *SMG3* (*smg3-2*, *smg3-3*) displayed similar defects, confirming the role of *SMG3* as a positive regulator of grain size.

**Figure:**
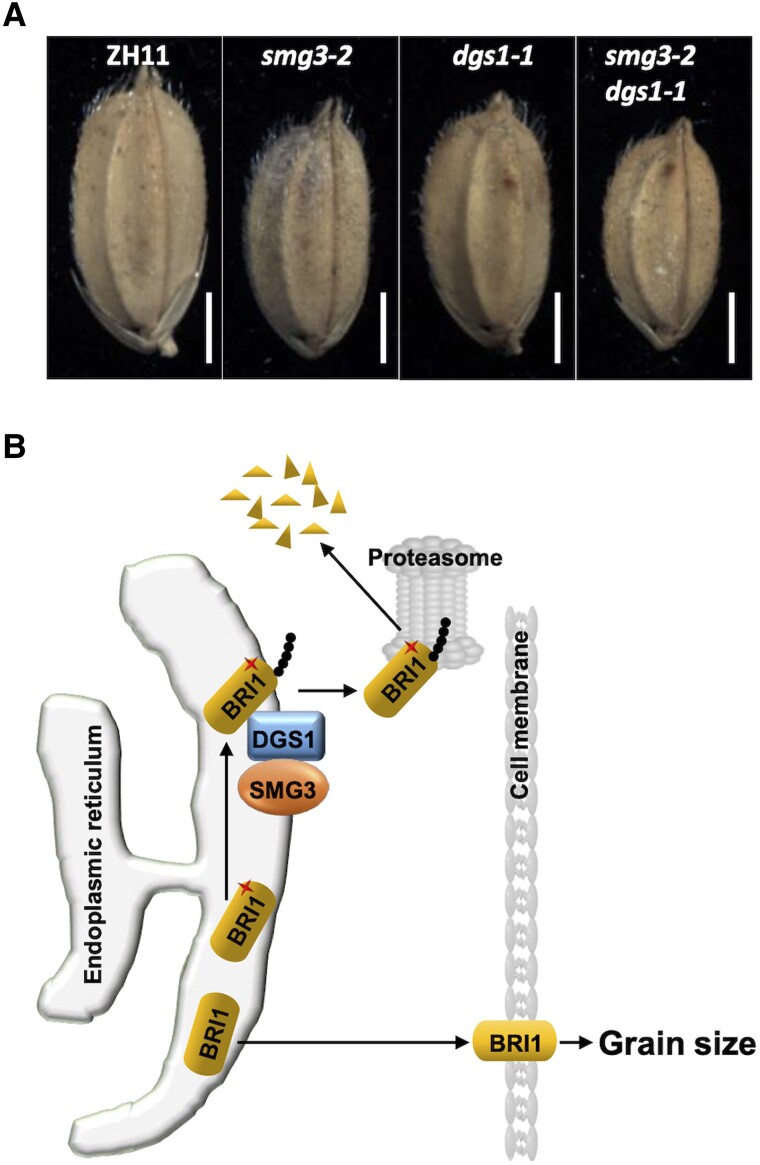
SMG3 and DGS1 influence grain size in rice. A, Grains of ZH11, *smg3-2*, *dgs1-1*, and *smg3-2 dgs1-1*. Scale bar, 2 mm. B, A model illustrating the participation of ERAD-related E2–E3 enzyme pair SMG3 and DGS1 in the degradation of misfolded BRI1 to regulate grain size. Modified from Figures 3 and 7 of Li et al. (2022).

*SMG3* is a homolog of the Arabidopsis Ubiquitin conjugase *UBC32*, an E2 ubiquitin-conjugating enzyme involved in the ERAD pathway (Cui et al., 2012). SMG3 in rice has a similar function since an E1 enzyme can modify SMG3; however, the protein generated by the *smg3-1* allele could not be modified, suggesting that SMG3 is a functional E2 enzyme. Analysis of a marker line (*GFP-SMG3*) revealed that SMG3 is localized to the endoplasmic reticulum where it colocalized with the ER-marker HDEL. At the whole plant level, the staining patterns of the *pSMG3::SMG3-GUS* showed that the *SMG3* gene is expressed in the developing panicles and the roots.

To identify other components in the ERAD pathway, the authors tested protein interactions between SMG3 and other known regulators of grain development in rice using yeast two-hybrid assays. The analysis indicated that SMG3 physically interacts with DECREASED GRAIN SIZE1 (DGS1), which has been previously characterized in the control of grain size regulation (Zhu et al., 2021). The SMG3-DGS1 protein interaction was confirmed in vitro with pull-down assays and in planta with split luciferase complementation assays. Further analysis using BIFC assays indicates that the SMG3-DGS1 interaction occurs in the ER. To analyze the effect of mutations in the *DGS1* gene, Li and colleagues generated four alleles in the ZH11 and KNK rice varieties (*dgs1-1*, *dgs1-2*, *dgs1-3*, and *dgs1-4*), which displayed defects in grain size. Interestingly, the double mutant *smg3-2 dgs1-1* showed a greater reduction in grain size than either single mutant alone. On the other hand, overexpression of *DGS1* in a ZH11 background resulted in longer rice grains. The defects in the *dgs1* mutant alleles resembled those of the *smg3* mutant. Interestingly, both *DGS1* and *SMG3* genes were expressed in developing panicles. At the subcellular level, analysis of the *GFP-DGS1* line indicates localization to the ER. To test if DGS1 is a functional E3 ligase, the authors expressed the protein in *E. coli* to conduct a biochemical analysis. Since a full DGS1 could not be expressed in *E. coli* they used a version lacking the transmembrane domain (DGS1ΔTM) to perform a ubiquitination assay. This analysis indicated that DGS1 can autoubiquitinate; and that mutations in the RING domain in DGS1 abolished the activity.

A previous study of the SMG3 homolog in Arabidopsis (UBC32) indicated that this protein participates in the degradation of the brassinosteroid receptor BRASSINOSTEROID INSENSITIVE 1 (BRI1), so authors tested the relationship between SMG3, DGS1, and BR signaling in rice. They found that the *smg3-1* and *dgs1* mutants were less sensitive to brassinosteroids. Additionally, the *smg3* and *dgs1* mutants had changes in gene expression consistent with changes in BR function (e.g. the expression of the *DWARF4* gene, involved in BR biosynthesis, was increased in the *dgs1* and *smg3*). In contrast, the expression of BR-signaling genes *BZR1* and *GSK2* was decreased. Furthermore, in vitro experiments demonstrated the interaction between BRI1 and DGS1, also found in planta with split luciferase assays. This interaction is critical since DGS1 was identified as a functional E3 ubiquitin ligase and can ubiquitinate BRI1.

To explore the effect of DGS1 on BRI1 function, the authors used an anti-BRI1 antibody to measure the accumulation of the BR receptor in different genetic backgrounds. The BRI1 protein levels were increased in the *dgs* and *smg3* mutants, suggesting that SMG3 and DGS1 affect the abundance and hence the function of the BRI1 receptor.

In summary, this work provides evidence for the participation of ER-associated degradation in plant hormone signaling in the control of reproductive development. Some interesting phenomena are still to be discovered regarding the involvement of SMG3 in the responses to abiotic stresses and its possible participation in root development.
